# Progress in Skin Diseases

**Published:** 1903-04-04

**Authors:** 


### Progress in Skin Diseases.

**Psoriasis.**—Malcolm Morris[1] gives a summary of recent work and opinion on this disease. The primary lesion, according to Munro, consists of an erosion on the horny layer of the epidermis in which a few leucocytes congregate, covered merely by some dislodged cells. Thus we get a small "dry" abscess at the surface of the horny layer, which is followed by hypertrophy of the layer and the formation of similar abscesses. The cause of this leucocytosis remains unknown, and there are objections to all the current theories. The disease is rarer in America than in Northern Europe, and in negroes than in whites. The lesions often disappear in wasting diseases such as cancer, and in fevers. Nerve disorders, such as asthma and intestinal troubles are probably a result of toxæmia from the injured skin rather than a cause of the disease. Occasionally it appears with rheumatoid arthritis, and here we find marked constitutional symptoms. In acute cases arsenic is injurious, and in healthy persons only local treatment is desirable, such as the application of chrysarobin, tar, pyrogallic acid, and mercury. If rheumatoid arthritis is present, arsenic, with a diet of raw meat and hot water is of great value. Leslie Roberts thinks that the disease is due to a chemical irritant, possibly the product of some gland such as the thyroid. Pernet advocates salicyn in large doses except in very old chronic cases. Eddowes recommends daily painting with liquor picis carbonis till the part is inflamed or the scales become thick, and then boric ointment and hot water for two or three days. Gilchrist finds 40 grains of iodide of potash thrice daily and salicylic acid externally very useful. Balzer[2] recommends treatment with chrysophanic acid for a fortnight after removal of the scales by baths and ointments, but no alkaline applications should be used while chrysophanin is being given, because the combination may set up a dermatitis. Next, pure oil of cade is applied either alone or diluted with starch. After a cure is effected oil of cade baths may be given for a time. He advises also a restricted diet and arsenic internally. Watson and Thompson[3] describe experiments showing the value of a product of bone marrow called myelocene in the treatment of psoriasis.

**Vaccine Rashes** are reviewed by G. Pernet[4] who notes a large number, such as delay of the formation of vesicles for months, second crops of vesicles; incidental rashes, such as urticaria, erythema of various forms, and lichen. Impetigo contagiosa and eczema may be easily grafted on to the sore and spread elsewhere. Generalised vaccinia may be due in some cases to transplantation of vaccine matter to sore points elsewhere. Morbilliform and scarlatiniform rashes are more common, and of no importance. Of course every rash appearing after vaccination is popularly ascribed to it; thus even the itch has been accounted for. It is well known that the danger of syphilitic infection was theoretically possible with human but not with calf lymph, though an unclean lancet might still convey it. Tuberculosis from calf lymph is extremely doubtful, and leprosy cannot be conveyed by inoculation so far as experiments have gone. Septic diseases, such as tetanus and erysipelas, may attack any scratch or open wound. Syphilis psoriasis and eczema, which are in a latent stage, may appear soon after vaccination and will be credited to it, but eczema is sometimes not aggravated and may occasionally even be cured by vaccination, and existing psoriasis does not seem to be affected by it. Whooping-cough seems to be benefited, nævi are in some cases removed by it, and the course of leprosy has been checked according to the observations of Beavan Rake.

**Gelatine Dressings.**—A. Eddowes[5] discusses the mode of preparation and application of these dressings and the class of cases for which they are suitable. One of the most useful is composed of zinc oxide one part, gelatine two parts, glycerine three, and water four parts. The gelatine is first soaked in some of the water for several hours and then the rest of the ingredients mixed by the aid of heat. When cold it can be cut into blocks and kept in a well-corked bottle till required. It is then dissolved in a jam pot placed in boiling water and painted on the skin. To strengthen it a fine layer of cotton wool may be pressed on to it while wet, or gauze bandages may be placed around it. It is a drying and cooling dressing, very flexible, causes no chafing or itching, can be cut away or patched, or removed in a minute by hot water. Thus for the neck, face, or limbs, as well as the trunk it forms a most comfortable and lasting application, but on account of the feeling of coolness rather extra clothing is needed. He insists that the skin should be first cleansed with spirit, and then dusted with a powder of calomel one part and starch four parts. The spirit must dry off before the film is painted on. The treatment is invaluable for varicose eczema and the dressings need not be removed oftener than once a week or even much longer if the skin is well disinfected first. In eczema, burns, herpes, or dermatitis herpetiformis it forms a very useful dressing, and in ringworm chrysarobin, or other drugs may be mixed with it and bandages discarded.

**Treatment of Itching.**—Hartzell[6] says that sometimes diet, or general remedies, such as bromides and antipyrin, are useful, and where the skin is very dry, pilocarpine (gr. $\frac{1}{20}$ in pills three times daily). For local remedies carbolic lotion or ointment or weak menthol ointments are excellent. In moist eczema recourse may be had to resorcin with $\frac{1}{2}$ per cent. of sodium chloride—*e.g.*, resorcin gr. $7\frac{1}{2}$, sodii chloridi gr. $3\frac{1}{2}$, glycerini dr. 2, liquoris calcis 1 oz. Another remedy, especially useful in senile pruritus is thymol gr. $\frac{1}{2}$–1 to the ounce, with or without a little liq. potassæ. Liquor carbonis detergens, 10 to 30 drops to the ounce, relieves the irritation of eczema. Hutchinson says that if it fails to give relief a weaker solution should be tried. For itching about the perineum hydrargyrum perchloridum gr. $\frac{1}{2}$ to the ounce may be applied, and for pruritus vulvæ we may paint every second day with nitrate of silver 10–60 grains to the ounce, or with spirits of nitrous ether, or with the mixture of camphor and chloral. Cocaine of course gives temporary ease.

**Icthyol.**—Eddowes[7] says that when given for acne rosacea internally the liquid form should be preferred, so as to act on the tongue and teeth antiseptically if there is caries or tartar. When patients cannot bear the nauseous flavour, pills must be substituted with some disinfecting gargle. Externally it is of great value in acne, erythema, erysipelas, urticaria, and in eczema rubrum of the legs it can be applied as a lotion on bandages in the strength of 1 to 5 or 10 per cent; or it can be painted on the legs and a zinc gelatine plaster applied over it. Its efficacy here seems due to its reducing congestion and destroying the streptococci on the skin.

**Ringworm.**—A. W. Mewborn[8] allows that the ordinary microsporon affects only the scalp, and that in children alone, but he shows that perplexing cases exist due to the microsporon of the cat. Thus there may be small spored forms in the scalp, and large chain-like ones on the body, due to the same infection. When this organism is grown on glucose agar a large flat circular growth appears, with concentric rings and a marginal fringe lying at a tangent to the left like the teeth of a circular saw. When growing around hairs it shows at the earliest stage large jointed mycelia, which soon break up into a mass of spores. The writer refers to the importance of not using ointments with an oily base, as the oil renders them harmless to the ringworm organisms. The remedy should be in aqueous or spirituous solutions. Thus salicylic acid in alcohol or ether is quickly fatal. Equal parts of glycerine and tincture of iodine, or guttapercha solution containing chrysarobin 10 per cent., and salicylic acid 5 per cent. may be employed after the skin has been cleansed from fat and the diseased hairs epilated.

**Tuberculides.**—T. Colcott Fox[9] in classifying tuberculous eruptions remarks that besides miliary tuberculous ulcers with numerous bacilli, lupus vulgaris, and the necrotic scrophulo-derma, we sometimes find tuberculous gummata in children and disseminated lupus after fevers in childhood, and a group of tuberculous eruptions such as lichen scrophulosorum, various papular and nodular eruptions, and erythema induratum. Some of these are scaly, some pustular or necrotic, and others ulcerative. In the lichen type we have a number of very small indolent papules which appear suddenly on the trunk, limbs, and particularly on the lower part of the trunk in young persons, and which tend to vanish after an indefinite period. The erythema induratum or Bazin's disease, is found on the calves of the legs and occasionally on the back of the hands, like a gumma; it gradually destroys the skin over it, and may break down again and again through many years. As to the other forms, though the appearances are varied, they show the initial cellular infiltration, the plasma cell mass; they appear suddenly, cause few sensory symptoms, are bilateral in situation, and undergo various changes, such as atrophy or suppuration. The evidence of their tuberculous origin is strong, though the bacillus has rarely been found in them. Some writers have ascribed them to the effects of tubercular toxin, or dead bacilli, or to compound infections, but the pathology remains doubtful. Graham Little[10] points out that Bazin's disease is most common in young and anæmic girls. It may be accompanied by other typical tubercular diseases, reacts to tuberculin, and has caused tuberculosis when the tissues have been injected into guinea-pigs, though the bacillus has not yet been found in the disease itself. Darier's term "tuberculides" applies only to lesions in which the bacillus has not been found, so that if it is discovered the disease in question must be transferred strictly speaking to the true tubercular class. Fox gives the name of acne scrophulosorum to a type seen in weakly children. The eruption is indolent and scattered about the limbs, especially on their external aspects and on the buttocks. It consists of small papule pustules acuminate in the early stages. The purulent head shells out and leaves a crater, and the papule finally takes the form of lichen, leaving stains or faint scars. The patients often show other tuberculous lesions, and the eruption may disappear under a course of cod-liver oil.

[1] Brit. Med. Jour., Oct. 25. [2] Ibid., Dec. 27. [3] Lancet, Oct. 18. [4] Ibid., Jan. 10. [5] Med. Times, Sept. 27 [6] Therap. Gaz, 1902, No. 2. [7] Med. Times, Aug. 30. [8] New York Med. Jour., Nov. 15. [9] Clin. Jour., Sept 3. [10] Ibid., Jan. 27.

